# Population-specific genomic risk markers for oral and maxillofacial malignancies in a high tobacco-exposure cohort from Eastern Uttar Pradesh, India

**DOI:** 10.6026/973206300221433

**Published:** 2026-03-31

**Authors:** Pavan Kumar Dubey, Neelam Mittal, Gyaneshwar Chaubey, Rahul Agarwal, P.G Naveen

**Affiliations:** 1 Department of Prosthodontics and Crown & Bridge, Faculty of Dental Sciences, Institute of Medical Sciences, Banaras Hindu University, Varanasi, Uttar Pradesh, India; 2 Department of Conservative & Endodontics, Faculty of Dental Sciences, Institute of Medical Sciences, Banaras Hindu University, Varanasi, Uttar Pradesh, India; 3 Department of Zoology, Institute of Science, Banaras Hindu University, Varanasi, Uttar Pradesh, India

**Keywords:** Oral squamous cell carcinoma (OSCC), mitochondrial DNA polymorphisms (mtDNA), whole exome sequencing (WES), population-specific variants, tobacco-associated malignancy, eastern Uttar Pradesh

## Abstract

Oral squamous cell carcinoma (OSCC) dominates oral malignancies in Eastern Uttar Pradesh, India, driven by high smokeless tobacco use and late-stage presentations causing substantial mortality. Hence, this case-control study integrated mitochondrial DNA (mtDNA) profiling and whole exome sequencing (WES) in 98 participants (47 OSCC cases, 51 matched controls) from this high-burden region. mtDNA analysis revealed significant depletion of the 16223C polymorphism in cases (53.2% vs 90.2% controls; χ^2^=16.74, p=0.0001), indicating potential protective effects against OSCC development. WES identified case-enriched driver variants, including NOTCH1 p.R2156H (15.0% cases) and truncating FAT1 mutations (10.0% cases), highlighting key oncogenic pathways. These findings advance precision oncology by establishing population-specific genomic markers for risk-stratification and targeted prevention of tobacco-related OSCC in Northern India.

## Background:

Oral squamous cell carcinoma (OSCC) is the most common histopathological variant of oral and maxillofacial malignancies, constituting about 90-95% of all oral cavity malignancies in the global arena [1]. The burden of lip and oral cavity cancers has shown a significant rise all over the world, with almost 370,000 new cases and 199,000 deaths reported in 2019, which is a 109.6 per cent rise in the number of new cases since the year 1990 [2]. This growing load is highly disproportionate to low and middle-income nations, especially those in South and Southeast Asia, where consumption habits of tobacco vary drastically from those in the West [3]. India has a remarkably large share of the world OSCC burden of about 25-33% of the global burden and about 77,000-80,000 new cases of OSCC are estimated every year [4]. Age-standardised incidence rate of oral cancers in India (9.32 per 100,000) is significantly higher compared to those in the world, with five-year survival rates being dismal at 20-45 per cent as oral cancers are presented mostly at advanced stages [5]. This epidemiological trend indicates the distinctive etiological situation of oral cancers in India with smokeless tobacco products of gutkha, khaini, paan masala and zarda as the most frequent of the carcinogenic exposures, rather than the smoking and alcohol-dominated aetiology of western cohorts [6]. Eastern Uttar Pradesh (EUP), which is a region with densely populated districts such as Varanasi and Gorakhpur, is one of the regions in India with the highest burden of oral malignancies [7]. The database of the Varanasi district on population-based cancer registries records mouth and tongue cancers as the top sites of malignancy among males, with an age-adjusted incidence rate of 22.4 per 100,000 in the urban community and a cumulative lifetime risk of 1 in 48 years of age among males in the 0-74 age group, which is the second highest in India [8]. Prevalence of tobacco use in Uttar Pradesh (35.5%) is far higher than the national prevalence of tobacco use (28.6%) and the population-attributable fraction in oral malignancies of smokeless tobacco is 57.5% in South Asian persons [9]. Genome studies have made significant contributions to the study of the pathogenesis of OSCC. Recurrent TP53, NOTCH1, FAT1, CASP8, PIK3CA and CDKN2A driver mutations have been identified in extensive genomic characterisation programs such as The Cancer Genome Atlas (TCGA) and International Cancer Genome Consortium (ICGC) as recurrent mutations in head and neck squamous cell carcinomas [10]. A database of Indian OSCC cohorts named dbGENVOC has been established to catalogue variants found in Indian OSCC cohorts and has shown population-specific mutation patterns, such as higher levels of CASP8 variants and tobacco-specific mutations [11]. The implication of regional carcinogenic exposures on mutational landscapes was revealed by recent multimodal profiling of 261 OSCC genomes in India, whereby foundations of betel quid-associated changes in analogous pathways were identified [12]. Changes in mitochondrial DNA (mtDNA) have been suggested to play major roles in the pathogenesis of oral carcinogenesis. The fact that the mitochondrial genome is an easy target for oxidative damage, with scarce repair, makes it especially vulnerable to tobacco-induced mutagenesis [13]. D-loop defects have been observed in about 27 per cent of all OSCC cases and certain polymorphic mutations have been linked to tumour progression, metastatic potential and response to therapy [14]. Moreover, the cisplatin responsiveness of the variants is supported by the presence of the associations between the haplogroups of the mtDNA and cisplatin [15]. Although these have been made, there still exist considerable gaps in the nature of the genetic arrangement of tobacco-related OSCC among the Northern Indian populations. Earlier genomic research has been restricted mostly to Southern Indian populations or has used small-scale coverage methods, which are not sufficient to detect rare variants of populations [16]. The history and demographics of Northern India are unique, with different admixtures and possibly founder effects, which require region-specific studies to determine the variants of susceptibility that are likely under represented or missing in the global databases [17]. Moreover, using mitochondrial and nuclear genomic data to comprehend epistatic interactions in tobacco-exposed populations is not a field that has been properly investigated [18]. Therefore, it is of interest to determine population-specific genomic risk signals of oral and maxillofacial malignancies in the high tobacco-exposure cohort in Eastern Uttar Pradesh by addressing the combined methodologies of global genome analysis, mitochondrial DNA profiling and whole exome sequencing to explain common and rare variants which contributed to modifying the susceptibility to the disease in this high-burden area.

## Materials and Methods:

## Design and ethical concerns of the study:

The case-control study took place in the Faculty of Dental Sciences, Banaras Hindu University, Varanasi, India, in the period between January 2020 and December 2023.

## Population and sample size of the study:

Ninety-eight participants were recruited, including 47 histopathologically defined OSCC patients and 51 age, sex and tobacco-exposure- matched healthy controls that lived in the same geographic area. Also, 40 samples (20 cases, 20 controls) were chosen and subjected to whole-exome sequencing depending on the presence or absence of mitochondrial DNA mutations and clinical variables. In the analysis of the global population structure, 383 publicly available repositories with high-coverage whole genomes of diverse populations were analysed.

## Inclusion criteria:

All the cases were of adult patients (knowledge of age of 18 years) with primary oral cavity squamous cell carcinoma of tongue, buccal mucosa, mandibular alveolus, floor of mouth, hard palate or retromolar trigone verified by histopathological examination using incisional or excisional biopsy. A history of tobacco consumption (smoke or smoke less, such as gutkha, khaini, zarda, or bidi) and/or harmful dietary practices (areca nut/betel quid chewing) was required to be included. It was restricted to the population of Eastern Uttar Pradesh or neighbouring North Indian areas and the presence of fresh/frozen tumour tissue and matching normal blood or saliva samples that were sequencing-able. Control was in terms of age, gender and tobacco habit-matched healthy people who have not had malignancy, premalignant lesions (leukoplakia, oral submucous fibrosis, erythroplakia), or suspicious oral mucosal changes. Control was recruited within the same geographic and socioeconomic strata as cases.

## Exclusion criteria:

Patients with the malignancies of non-oral origin (oropharynx, hypopharynx, larynx, skin or oesophagus), metastatic carcinoma of unknown origin, recurrence or recurrence of pre-existing malignancies, or a history of any cancer were excluded. Those who received any neoadjuvant therapy (chemotherapy or radiotherapy) before sample collection were excluded to prevent artefacts due to therapy. Other non-SCC histologies, such as verrucous carcinoma not associated with tobacco, mesenchymal tumours, lymphomas, or salivary gland malignancies, were eliminated. Cases with no history of documented tobacco/deleterious habit, where other etiologies like HPV-positive tumours in the absence of tobacco history were assumed as the main causes and where tissue quality was not adequate or clinical data were not recorded fully, were also excluded. Pediatric patients (under the age of 18 years) and patients with syndromic links (Fanconi anaemia, xeroderma pigmentosum) were excluded.

## Sample collection and DNA extraction:

All the subjects donated peripheral venous blood samples of 5 mL in EDTA-coated vacutainers. In the case of OSCC, tumour tissue samples were collected either through diagnostic biopsy or surgical resection and placed directly at -80°C. Blood and tissue samples were extracted with the QIAamp DNA Blood Mini Kit (Qiagen, Hilden, Germany) accordingly and the QIAmp DNA FFPE Tissue Kit, respectively, in accordance with the manufacturer's protocols. NanoDrop spectrophotometry (A260/A280 ratio 1.8-2.0) was used to determine the quality of the DNA and Qubit fluorometry was used to determine the quantity of the DNA.

## Global genome analysis:

To establish a baseline of population structure, 383 high-coverage whole genomes of the Estonian Biocentre Global Reference Panel, which is a collection of geographically diverse populations, such as South Asian, European, African and East Asian, were analysed. Joint GRCh38 reference-aligned GVCFs were used in variant calling. Annotation was performed using VEP and ANNOTAVAR to assess the impacts of functional results and prioritise the cancer-associated genes. Population structure was described by principal component analysis and admixture modelling, whereas fixation index (FST) was used to determine loci undergoing selection or differentiation in populations.

## Mitochondrial DNA sequencing:

Minimal parts of both ends of the entire mitochondrial genome (16.6 kb) were amplified in an overlapping long-range PCR reaction. Preparation of libraries was done according to Illumina TruSeq protocols and they were sequenced on the Illumina NovaSeq 6000 platform with paired-end sequencing of 150 bp, designed to achieve a minimum coverage of 10,000x to detect heteroplasmy at a minimum threshold of >1%. BWA-MEM was used to align the sequence to the updated Cambridge Reference Sequence (rCRS, NC_012920.1). Mutect2 was used in variant calling with the estimation of heteroplasmy of somatic mutations using GATK. The assignment of the Haplogroup was done using HaploGrep2 software and Phylotree Build 17.

## Whole exome sequencing:

Exome capture was done on the Agilent SureSelect Human All Exon V6 kit that has about 60 Mb of coding regions. Illumina NovaSeq 6000 was sequenced on 2x150 bp paired-end reads and this produced a mean depth of more than 100x. The GATK best practices were observed in bioinformatic processing, alignment with BWA-MEM to GRCh38, duplicate marking with Picard and variant calling with HaplotypeCaller to label germline variants and Mutect2 to label somatic variants. Variant filtration selected high pathogenicity score (CADD>20) variants with low frequency (MAF less than 0.01 in gnomAD v3.1.2). SIFT, PolyPhen-2 and Mutation Taster were used to predict pathogenicity by functional prioritisation and OncoDriveCLUST and MutSigCV were used to identify driver genes.

## Statistical analysis:

Descriptive statistics were used to summarise demographic and clinical data: means and standard deviations of continuous data, frequencies and percentages of categorical data. Group comparisons used independent samples t-tests in case of continuous variables and Chi-square tests or Fisher's exact tests in case of categorical variables. In the case of the mitochondrial DNA association studies, 2x2 contingency tables were created on each polymorphism, Chi-square tests were done and a Bonferroni correction was used to control multiple testing (p=.05/91=.00055 with 91 common polymorphisms analysed). The logistic regression was adjusted on the basis of age, sex and the duration of tobacco exposure and compared the haplogroups. Permutation testing of 10,000 iterations was used to test differences in overall mutation burden. Significant associations were found to have odds ratios with 95 per cent confidence intervals. In the whole exome sequencing data, the frequencies of variants were compared between the cases and controls using Fisher's exact test. Pathway enrichment analysis has used the KEGG and Reactome databases. R version 4.2.0 and PLINK v1.90 were used to carry out all statistical calculations. The statistical significance was defined as p< 0.05, unless otherwise specified, to correct multiple testing.

## Results:

The demographic and clinical profiles of study participants are presented in Table 1 (see PDF). The case cohort demonstrated significant male predominance with 40 males (85.1%) and 7 females (14.8%), yielding a male-to-female ratio of 5.7:1. Mean age at diagnosis was 52.4 ± 12.3 years (range: 32-78 years), with peak incidence in the fifth and sixth decades. Occupational analysis revealed that 34 cases (72.3%) were employed as farmers or manual labourers, professions associated with prolonged tobacco exposure and limited healthcare access. Tobacco exposure patterns demonstrated smokeless tobacco as the predominant form (68.1%, n=32), followed by smoking (21.3%, n=10) and combined habits (10.6%, n=5). Mean duration of tobacco use was 14.7 ± 6.8 years, with 63.8% reporting usage exceeding 10 years. Among smokeless tobacco users, gutkha was the most commonly consumed product (56.3%), followed by khaini (28.1%) and zarda (15.6%). Anatomical distribution showed buccal mucosa as the most common primary site (44.7%, n=21), followed by tongue (34.0%, n=16), mandibular alveolus (14.9%, n=7) and other sites (6.4%, n=3). Histopathological grading classified cases as moderately differentiated SCC (53.2%, n=25), well-differentiated SCC (38.3%, n=18) and poorly differentiated SCC (8.5%, n=4). Clinical staging revealed advanced disease in 78.7% of cases: Stage III (55.3%, n=26) and Stage IV (23.4%, n=11), with only 21.3% presenting at early stages (Stage I-II, n=10). Control participants were adequately matched for age (mean 50.8 ± 11.7 years; p=0.523), sex distribution (82.4% male; p=0.715) and tobacco exposure patterns (68.6% smokeless; p=0.956), with comparable duration of tobacco use (13.2 ± 5.9 years; p=0.248).

Analysis of 383 whole genomes demonstrated distinct population clustering through principal component analysis, with South Asian populations forming a discrete clade intermediate between West Eurasian and East Asian clusters. Northern Indian samples positioned within the South Asian cluster showed evidence of West Eurasian admixture consistent with historical migration patterns. Fixation index (FST) calculations identified elevated differentiation at established OSCC driver genes between South Asian and global populations: TP53 (FST=0.12), NOTCH1 (FST=0.15), FAT1 (FST=0.10) and CASP8 (FST=0.11). South Asian-specific allele frequencies for NOTCH1 variants (0.08) exceeded global averages (0.03), suggesting population-specific enrichment. Pathway enrichment analysis highlighted amplified signals in cell cycle dysregulation and PI3K-AKT signalling pathways within South Asian subsets, potentially reflecting chronic carcinogen exposure effects. Mitochondrial genome sequencing identified 315 distinct polymorphisms across all samples. Among 91 common polymorphisms analysed in the hyper variable segments (HVS-I and HVS-II), three demonstrated differential distribution between cases and controls, as detailed in Table 2 (see PDF). The 16223 polymorphism (T>C transition in HVS-I) showed significant depletion in cases compared to controls: present in 25/47 cases (53.2%) versus 46/51 controls (90.2%), yielding χ^2^=16.74, df=1, p<0.0001. This association remained highly significant after Bonferroni correction (α=0.00055). The odds ratio for OSCC in the absence of the 16223 polymorphism was 8.10 (95% CI: 2.83-23.17), suggesting a protective effect of this variant. Polymorphisms 16126 (C>T) and 16311 (T>C) demonstrated nominal case enrichment but failed to achieve statistical significance after correction. The 16126 variant was present in 8/47 cases (17.0%) versus 4/51 controls (7.8%; χ^2^=1.90, p=0.17). The 16311 variant was present in 12/47 cases (25.5%) versus 6/51 controls (11.8%; χ^2^= 3.10, p=0.08). Overall, mitochondrial mutation burden was marginally higher in cases (mean 4.43 ± 1.67 mutations per individual) compared to controls (mean 4.00 ± 1.52), with a difference of 0.43 mutations. However, permutation testing (10,000 iterations) indicated this difference was not statistically significant (p=0.28). Haplogroup analysis confirmed macro-haplogroup M predominance in both groups (cases: 82.9%, controls: 84.3%; p=0.854), consistent with Indian subcontinent ancestry. Sub-haplogroup M5a showed nominal depletion in cases (OR=0.62, uncorrected p=0.09), though this did not survive multiple testing correction. Heteroplasmy levels exceeding 1% were rare (1.2% of all variants) with no significant enrichment in tumour-derived samples.

Regional haplogroup origin analysis revealed significant differences between cases and controls (χ^2^=10.28, p=0.0013). Cases demonstrated overrepresentation of West Eurasian (WEU) haplogroups (13/47, 27.7%) compared to controls (1/51, 2.0%; expected 6.71 and 7.29, respectively). Conversely, South Asian (SA) haplogroups were underrepresented in cases (33/47, 70.2%) compared to controls (49/51, 96.1%; expected 39.29 and 42.71, respectively). Whole exome sequencing of 40 representatives samples (20 cases and 20 controls), stratified by 16223 polymorphism status and clinical stage, generated approximately 45,000 variants per sample. Following rigorous filtration for rare and deleterious variants (MAF<0.01, CADD>20), case-enriched alterations were identified in established OSCC driver genes and novel population-specific loci, as shown in Table 3 (see PDF). Among established driver genes, NOTCH1 missense variant (p.R2156H; c.6467G>A) was detected in 3/20 cases (15.0%) and 0/20 controls (p=0.032; CADD score 28.4). FAT1 truncating mutations were identified in 2/20 cases (10.0%) with no control occurrences (p=0.078; CADD score 35.2). A novel frame shift variant in USP9X was detected in 1/20 cases (5.0%). Notably, three novel variants not previously reported in global OSCC databases (dbGENVOC, COSMIC, TCGA) or population databases (gnomAD, 1000 Genomes) were identified exclusively in cases: PLA2G2A (c.287G>A, p.R96H; 3/20 cases, 15.0%; CADD 24.7), POLE (c.1234C>T, p.R412W; 2/20 cases, 10.0%; CADD 26.3) and BRIP1 (c.2392C>T, p.R798C; 2/20 cases, 10.0%; CADD 25.8). These variants were classified as likely pathogenic by SIFT/PolyPhen-2 predictions. Integration with mitochondrial data revealed epistatic patterns: samples lacking the protective 16223 polymorphism demonstrated higher nuclear burden in oxidative stress pathway genes, with NFE2L2 pathway alterations present in 4/10 such cases (40%) compared to 1/10 samples with the 16223 polymorphism (10%). Pathway enrichment analysis confirmed PI3K-AKT signalling dominance among case-enriched variants, with tobacco-signature overlaps (C>A transversions) from global analysis data.

## Discussion:

The presented study is the complex genomic research of oral and maxillofacial malignancies in a high-exposure-to-tobacco cohort in Eastern Uttar Pradesh that combines the use of both mitochondrial DNA profiling and whole exome sequencing as the methods to determine the risk factors that are specific to the population. The results demonstrate different genomic architectures of tobacco-related OSCC in Northern India and the findings strongly associate numerous mitochondrial polymorphisms and nuclear variants that can be used to i nform precision prevention initiatives in this high-burden area. The important loss of the 16223 polymorphism of OSCC cases (53.2% vs 90.2% in controls; p<0.0001) is a new finding with significant implications on the role played by mitochondria in oral carcinogenesis. This change in hyper variable segment I TC transition has been earlier seen to be related to disturbed mitochondrial operation and oxidative stress reaction [19]. Our data indicate a protective effect (OR=8.10, disease no this variant) which can be attributed to the role of reactive oxygen species handling modulation in tissues exposed to tobacco, since mitochondrial dysfunction has been determined as a major pathway in smokeless tobacco-induced carcinogenesis [20]. Previous studies had reported D-loop mutations in an estimated 27 per cent of OSCC cases, but the particular polymorphisms varied across populations, highlighting the need to profile regions of the field specifically [21]. The prevalence of the macro-haplogroup M of our cohort (over 82% of the members in both categories) is in line with other patterns of Indian subcontinent ancestry [22]. But the strong overrepresentation of West Eurasian haplogroups of cases (27.7 vs. 2.0 per cent in controls) not only indicates that the susceptibility variants were potentially introduced by ancient admixture but also indicates that the susceptibility variants interact with tobacco exposure to increase the risk of cancer. This finding builds upon recent work in population genetics that has shown that Northern Indian populations have significant components of West Eurasian ancestry that can affect the disease susceptibility patterns [23]. The theoretical tendency toward the depletion of M5a sub-haplogroup in cases (OR=0.62) should be investigated in larger samples, especially in the light of correlations between haplogroups of the actual MtDNA and the therapeutic response, such as cisplatin sensitivity [124]. Lack of general mutation burden differences in cases and controls (permutation p=0.28) indicates that particular polymorphisms and not general mitochondrial instability contribute to risk in tobacco-related OSCC. This is contrary to research showing an increase in the content of the mtDNA and deletions in the squamous cell carcinomas of the head and neck of the Western population who are smokers [25], which could be indicative of a different aetiology between smoking and smokeless tobacco carcinogenesis. Our Northern Indian cohort of OSCC has known driver mutations that were enriched by whole-exome sequencing. The identified NOTCH1 missense mutation (p. R2156H) observed in 15% of the cases is congruent with the results of overall genomic characterisation of the gene, that show that NOTCH1 is among the most frequently mutated genes in head and neck cancers [26]. NOTCH1 is a tumour suppressor of squamous epithelia and its inhibition leads to the malignant transformation by dysregulating differentiation programs [27]. In the same way, accumulating evidence has implicated FAT1 in oral carcinogenesis by regulating Wnt/-catenin and Hippo-YAP signalling through FAT1 truncating mutations (10% of cases) [28]. The discovery of three new forms in PLA2G2A, POLE and BRIP1 genes is an important addition to the OSCC genetics since neither of these variants could be found in all databases searched worldwide. The PLA2G2A gene is the secreted phospholipase A2 of inflammatory reactions and phospholipid metabolism, whose roles in cancer progression are emerging [29]. The identified variant (p.R96H) in 15% of cases could modify the enzyme activity and inflammatory communication of the oral mucosa that undergoes tobacco carcinogen exposure. POLE mutations are typically related to ultramutated phenotype in different types of cancers and the identified p.R412W variant could also affect the fidelity of DNA replication and mutagenesis frequency [30]. BRIP1 is the gene that encodes a helicase, which interacts with BRCA1 and operates within the DNA repair pathways and has been confirmed to play a role in the hereditary cancer syndromes, such as breast and ovarian cancers [31]. New variants of BRIP1 identified in tobacco-related OSCC are indicative of possible interaction between hereditary DNA repair impairment and carcinogenesis induced by the environment. The observed epistatic interaction of the presence of mitochondrial 16223 polymorphism with the absence of 40% versus 10% of NFE2L2 changes in the nucleus indicates that mitochondrial-nuclear crosstalk contributes to the pathogenesis of OSCC. This observation is consistent with new evidence according to which mitochondrial dysfunction can provoke compensatory nuclear reactions and can affect nuclear genome stability [32]. The NFE2L2 (NRF2) pathway is a major controller of antioxidant homeostasis and its malregulation has been associated with cancer progression, as well as adverse reactions to treatment [33].

The analysis of the global population structure placed Northern Indian samples between the West Eurasian and East Asian clusters with high levels of fixation at the OSCC driver genes (NOTCH1 FST=0.15, TP53 FST=0.12), depicting the population differentiation at loci of disease interest. The enhanced PI3K-AKT signatures in South Asian subsets might be indicative of adapting to chronic exposure to carcinogens and are in line with epidemiological evidence of remarkably high burden of oral cancer in India [34]. These patterns highlight that the risk of people in the South Asian cohorts is not adequately reflected in the susceptibility variants found in the Western population and population-specific resources of genomic technology are needed. The demographic data of our cohort: a male majority (85.1%), the mean age of 52.4 years and advanced-stage presentations (78.7% Stage III-IV) and a prevalent involvement of the buccal mucosa (44.7) are consistent with the epidemiological trends in the region and confirm the representativeness of the sample [35]. The large percentage of farmers and labourers (72.3) indicates the exposure pattern of occupation in EUP, where the occupation of farmers and labourers has low healthcare access and low awareness, which leads to late presentations. Such socioeconomic conditions, in combination with genetic vulnerabilities, enhance the local cancer burden. There are a number of restrictions that should be considered. The small sample size (n=98 in the case of the mtDNA, n=40 in the case of WES) could be a constraint to power to detect rare variants and they might not capture the full genetic diversity of Northern India. This emphasis on tobacco-related cases, although suitable with such a high-exposure population, limits the ability to generalise to other etiologies such as HPV-related oropharyngeal cancers. Whole exome sequencing is, by nature, not sensitive to non-coding segments, which can harbour regulatory variants. In the lack of longitudinal follow-up data, the evaluation of the prognostic value of identified variants is not possible. Lastly, there was no functional validation of novel variants done in this study. The translational implication of these results is that there might be the development of inexpensive SNP/mtDNA panels that contain the 16223 polymorphism and other nuclear variants that could be used in risk stratification in populations that have high tobacco exposure. Genomic biomarkers can be incorporated with the current screening programs as part of the National Oral Health Programme to allow targeting of individuals at high risk. The discovery of population-specific variants that are not present in worldwide databases reinforces the importance of more genomic informatics representing populations across the South Asian continent to achieve the virtue of precision medicine in oral cancer.

## Conclusion:

We show population-specific genomic risk factors for tobacco-related oral squamous cell carcinoma (OSCC) in Eastern Uttar Pradesh, revealing distinct mitochondrial and nuclear contributions to disease susceptibility. The 16223 mitochondrial polymorphism demonstrates strong protective effects, complemented by case-enriched variants in driver genes NOTCH1 and FAT1, plus novel population-specific mutations (PLA2G2A, POLE, BRIP1). These findings establish a unique genomic architecture for Northern Indian OSCC absent in global databases, supporting targeted risk stratification and prevention strategies.
